# Bandwidth correction of Swarm GPS carrier phase observations for improved orbit and gravity field determination

**DOI:** 10.1007/s10291-021-01107-0

**Published:** 2021-03-09

**Authors:** Lucas Schreiter, Oliver Montenbruck, Franz Zangerl, Christian Siemes, Daniel Arnold, Adrian Jäggi

**Affiliations:** 1 Astronomical Institute, University of Bern, Sidlerstrasse 5, 3001 Bern, Switzerland; 2 DLR, German Space Operations Center, 82234 Oberpfaffenhofen, Germany; 3 RUAG Space GmbH, 1120 Vienna, Austria; 4 Delft University of Technology, Kluyverweg 1, 2629 HS Delft, The Netherlands; 5 GFZ German Research Centre for Geosciences, 14473 Telegrafenberg, Germany

**Keywords:** Orbit determination, Tracking loop, Loop filter, Ionospheric artifacts, Gravity field determination

## Abstract

Gravity fields derived from GPS tracking of the three Swarm satellites have shown artifacts near the geomagnetic equator, where the carrier phase tracking on the L2 frequency is unable to follow rapid ionospheric path delay changes due to a limited tracking loop bandwidth of only 0.25 Hz in the early years of the mission. Based on the knowledge of the loop filter design, an analytical approach is developed to recover the original L2 signal from the observed carrier phase through inversion of the loop transfer function. Precise orbit determination and gravity field solutions are used to assess the quality of the correction. We show that the a posteriori RMS of the ionosphere-free GPS phase observations for a reduced-dynamic orbit determination can be reduced from 3 to 2 mm while keeping up to 7% more data in the outlier screening compared to uncorrected observations. We also show that artifacts in the kinematic orbit and gravity field solution near the geomagnetic equator can be substantially reduced. The analytical correction is able to mitigate the equatorial artifacts. However, the analytical correction is not as successful compared to the down-weighting of problematic GPS data used in earlier studies. In contrast to the weighting approaches, up to 9–10% more kinematic positions can be retained for the heavily disturbed month March 2015 and also stronger signals for gravity field estimation in the equatorial regions are obtained, as can be seen in the reduced error degree variances of the gravity field estimation. The presented approach may also be applied to other low earth orbit missions, provided that the GPS receivers offer a sufficiently high data rate compared to the tracking loop bandwidth, and provided that the basic loop-filter parameters are known.

## Introduction

ESA’s three satellite mission Swarm was launched in November 2013 (Friis-Christensen et al. 2008). The satellites were placed in polar low Earth orbits with initial altitudes of 480 km (Swarm A, C) and 530 km (Swarm B) after the commissioning phase. The three satellites are equipped with geodetic-grade dual-frequency GPS receivers provided by Rüstungs Unternehmen AG (RUAG).

**Fig. 1:**
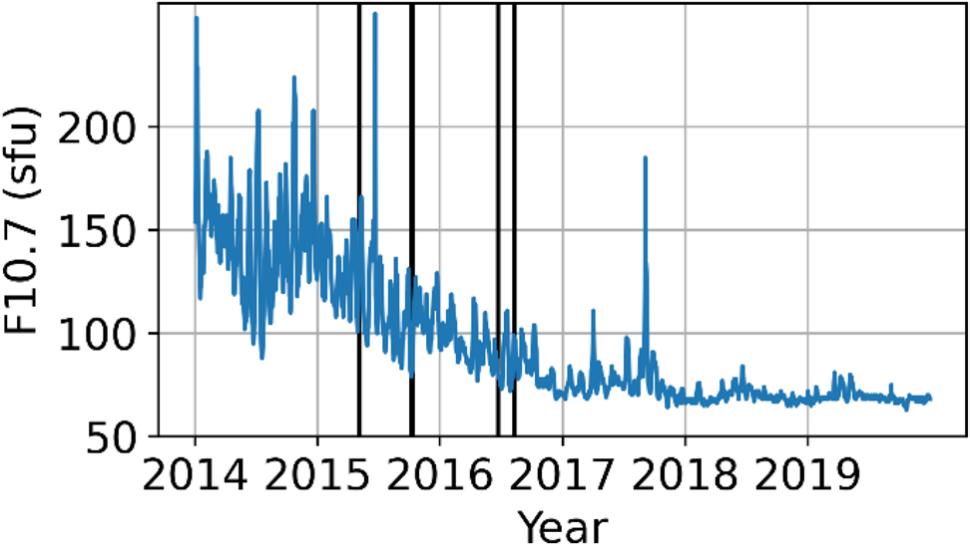
Solar flux index F10.7 over the first six years of the Swarm mission. Vertical black lines indicate the dates of the tracking loop updates summarized in Table 1

Due to the gap between the dedicated earth gravity field missions GRACE (Gravity Recovery And Climate Experiment; Tapley et al. 2004) and GRACE-Follow On, Swarm became a gap filler to provide monthly gravity field solutions (Lück et al. 2018). Especially in the first months of the mission, when the solar activity was relatively large, see Fig. 1, and during evening local times, artifacts in the gravity field became visible in Swarm gravity fields around the geomagnetic equator (Jäggi et al. 2016).

The GPS phase measurements leading to these artifacts can be identified using time-derivatives of the L1–L2 carrier phase difference. This difference is directly related to the slant TEC, i.e., the total electron content along the line of sight (Jäggi et al. 2016; Schreiter et al. 2019). Similar artifacts have already been observed for the GOCE (Gravity field and steady-state Ocean Circulation Explorer) Mission (Drinkwater et al. 2007; Jäggi et al. 2015), which used a different type of GPS receiver but identical chipset and tracking technique for semi-codeless tracking of the encrypted P(Y)-code.

After identifying bandwidth limitations of the carrier tracking as a likely cause of systematic measurement errors, the L1 and L2 phase locked loop (PLL) bandwidths were increased several times on the various satellites of the Swarm mission (Table 1; ESA 2015a, b, 2016). These updates had a positive impact on both precise orbit determination (POD) and gravity field determination as shown by van den IJssel et al. (2016) and Dahle et al. (2017) but could only benefit observations collected after their implementation. In a first effort, heuristic down-weighting schemes were applied to mitigate the impact of bad carrier phase observations during rapid changes in the L1–L2 carrier phase difference (Schreiter et al. 2019). To cope with this situation, the present study aims to develop a method for correcting carrier phase observations affected by bandwidth limitations. Based on a model of the loop filter provided by the receiver manufacturer, we assess the impact of rapid slant-TEC changes, and propose a method to recover the true L2 signal and to correct the L2 carrier phase observations. Eventually, we investigate the impact of these corrections on orbit and gravity field quality.

**Table 1:** Bandwidths of the phase locked loops for carrier phase tracking on the L1 frequency (*B*_L1_) and the L2 frequency (*B*_L2_) adopted in the Swarm GPS receivers in different mission periods (ESA 2015a, b, 2016; van den IJssel et al. 2016)

Since	Swarm-A	Swarm-B	Swarm-C
Launch	*B*_L1_ = 10 Hz, B_L2_ = 0.25 Hz	*B*_L1_ = 10 Hz, *B*_L2_ = 0.25 Hz	*B*_L1_ = 10 Hz, B_L2_ = 0.25 Hz
6 May 2015			B_L1_ = 15 Hz, B_L2_ = 0.50 Hz
8 Oct 2015	*B*_L1_ = 15 Hz, B_L2_ = 0.50 Hz		
10 Oct 2015		*B*_L1_ = 15 Hz, *B*_L2_ = 0.50 Hz	
23 June 2016			*B*_L2_ = 0.75 Hz
11 Aug 2016	*B*_L2_ = 0.75 Hz		*B*_L2_ = 1.00 Hz

We choose March 2015 and August 2015 for our tests to cover the narrowest L2 loop bandwidths (Table 1) with the most significant loop-related tracking errors and also to allow for direct comparison of different loop settings. Since all three Swarm satellites had the same loop settings in March 2015, we focused on Swarm A in this month. This satellite is part of the lower pair and thus affected by a larger slant-TEC than Swarm B. In August 2015, the L2 bandwidth for Swarm C was already updated to 0.5 Hz, but for Swarm A it was still at 0.25 Hz. Since Swarm A and Swarm C fly in close formation, they observe almost the same slant-TEC, which allows for a direct comparison of the tracking performance. For the computation of the orbits and phase residuals as well as the gravity field solutions, the development version of the Bernese GNSS software v5.3 was used (Dach et al. 2015).

## Models

This section discusses the origin of bandwidth-related phase tracking errors in the Swarm GPS receiver and provides the mathematical models for their correction. The loop filter response is analyzed in frequency-domain and a loop filter specific transfer function estimated. Eventually the transfer function is used to invert the loop filter.

### L2 tracking model and correction

Apart from constant offsets, the L1 and L2 carrier range ${\phi }_{1}, {\phi }_{2}$ may be split into the sum of a frequency-independent geometry term *g*(*t*) and a frequency-dependent ionospheric term, denoted as *I*(*t*) for the L1 carrier frequency *f*_1_. The impact of the ionospheric contribution on ${\phi }_{2}$ may be expressed as $\left({f}_{1}^{2}/{f}_{2}^{2}\right)I\left(t\right)$ in a first order approximation neglecting the higher-order ionospheric terms:

1 $$\phi_{1} = g\left( t \right) - I\left( t \right)$$

2 $$\phi_{2} = g\left( t \right) - \left( {\frac{{f_{1}^{2} }}{{f_{2}^{2} }}} \right)I\left( t \right).$$

In a time domain representation, the measured phase $\widehat{\varphi }\left(t\right)$ is obtained by convolution of the input phase $\phi \left(t\right)$ with the loop specific transfer function $H(t)$:

3 $$\hat{\phi }\left( t \right) = H\left( t \right){*}\phi \left( t \right).$$

Alternatively, the Fourier transform $\widehat{\Phi }\left(f\right)$ of the measured carrier phase range is given by the product

4 $${\hat{\Phi }}\left( f \right) = H\left( f \right) \cdot {\Phi }\left( f \right)$$

of the frequency-domain transfer function $H\left(f\right)$ and the Fourier transform of the true carrier range $\Phi (f)$. In case of L1 carrier phase tracking the tracking loop bandwidth is considered to be sufficiently high for both, the geometric and the ionospheric variation. Accordingly, the measured L1 phase range closely matches the true range:

5 $$\hat{\phi }_{1} \left( t \right) = H_{1} \left( t \right){*}g\left( t \right) - H_{1} \left( t \right){*}I\left( t \right) \approx g\left( t \right) - I\left( t \right) = { }\phi_{1} \left( t \right).$$

For the much smaller L2 bandwidth, this assumption does not hold. To be insensitive to geometry-related signal dynamics, the L2 PLL is therefore aided using the L1 carrier rate. The L2 carrier phase tracking may thus be described as

6 $$\hat{\phi }_{2} \left( t \right) = \hat{\phi }_{1} \left( t \right) + H_{2} \left( t \right){*}\left( {\phi_{2} \left( t \right) - \hat{\phi }_{1} \left( t \right)} \right),$$

which expands to

7 $$\hat{\phi }_{2} \left( t \right) = g\left( t \right) - I\left( t \right) + \left( {1 - \frac{{f_{1}^{2} }}{{f_{2}^{2} }}} \right) \cdot H_{2} \left( t \right){*}I\left( t \right).$$

The L2 tracking error is therefore given by

8 $$\hat{\phi }_{2} \left( t \right){ } - { }\phi_{2} \left( t \right) = { } - \frac{{f_{1}^{2} - f_{2}^{2} }}{{f_{2}^{2} }} \cdot \left( {H_{2} \left( t \right){*}I\left( t \right) - I\left( t \right)} \right).$$

To recover the true L2 carrier phase measurement, we apply ${H}_{2}^{-1}\left(t\right)$ to (6) and obtain

9 $$\phi_{2} \left( t \right) = \hat{\phi }_{2} \left( {t } \right) + \left[ {H_{2}^{ - 1} \left( t \right)*\hat{\phi }_{{{\text{gf}}}} \left( t \right) - \hat{\phi }_{{{\text{gf}}}} \left( t \right)} \right],$$

with ${\phi }_{\text{gf}}={\phi }_{2}-{\phi }_{1}$ denoting the geometry-free L1/L2 carrier phase combination. The term in square brackets describes the error of the L2 carrier phase and can be evaluated based on knowledge of the loop transfer function and the measured L1–L2 carrier phase difference.

The inversion is best performed in the frequency domain, where the inverse transfer function is given as ${H}_{2}^{-1}\left(f\right)=1/{H}_{2}(f)$. It should be mentioned that a GPS carrier phase measurement as provided, for example, in RINEX (Receiver Independent Exchange format; IGS 2019) observation data files is not the direct output of the tracking process. Instead, it is formed from the output of a numerically controlled oscillator (NCO), which is controlled by the actual tracking loop to follow a down-converted version of the received GPS signal. However, due to linearity of the required transformation and the properties of higher-order loop filters, Eq. (9) is not only valid for the measured NCO phase but can likewise be applied to RINEX carrier phase measurements.

### Swarm tracking loop

**Fig. 2:**
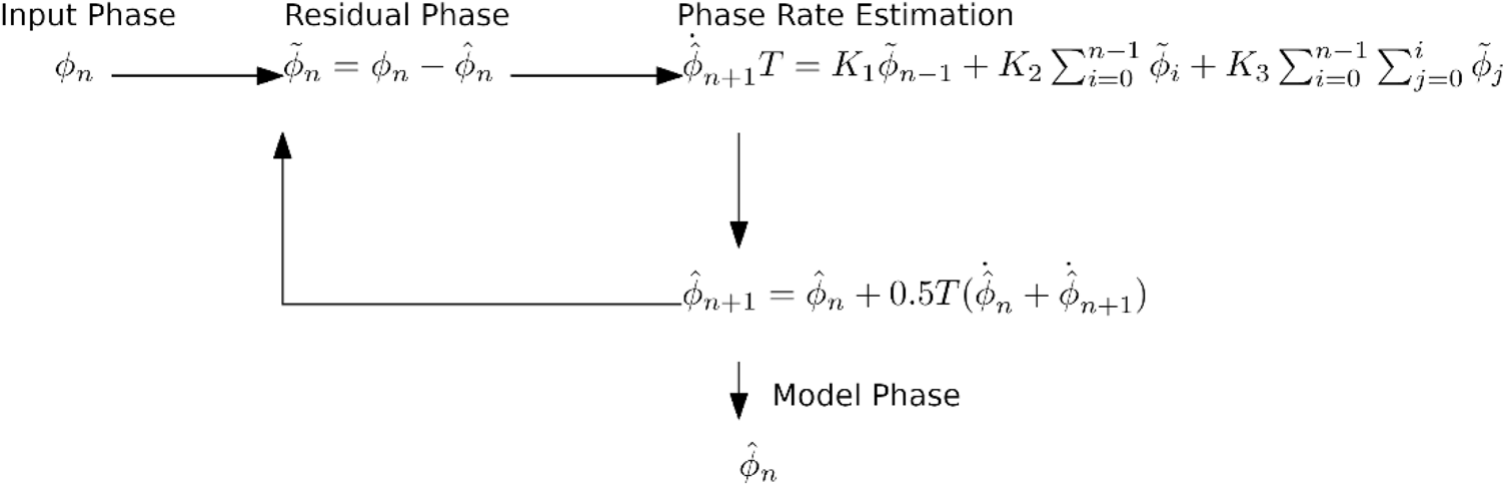
Simplified tracking processor with loop-filter, adapted from Thomas (1998)

According to personal information obtained from the manufacturer, the Swarm GPS-receivers use a Digital Phase Lock Loop (DPLL) of order 3 with rate-only feedback. An integration interval *T* = 0*.*01 s is used for the L1 carrier tracking, which is based on correlation with the open C/A-code signal. In contrast, a value of *T* = 0*.*1 s is used for the L2 phase measurement to compensate the incerased noise of the semi-codeless P(Y)-code tracking. Also, the PLL bandwidth for L1 is significantly wider than that for L2 (10–15 Hz vs. 0.25– Hz). By aiding the L2 tracking with the phase-rate from the L1 tracking, the capability to follow rapid signal variations associated with the dynamical motion of a satellite in low earth orbit (LEO) can, nevertheless, be retained despite the low L2 bandwidth. The tracking loop implementation involves a one-step delay between estimation and use of the phase rate and is described in Fig. 2.

The formulation itself is given by Stephens and Thomas (1995). We assume an ideal phase extractor. The *n*-th model residual phase ${\stackrel{\sim }{\phi }}_{n}$ is given by

10 $$\tilde{\phi }_{n} = \phi_{n} - \hat{\phi }_{n} ,$$

where ${\phi }_{n}$ is the measured phase, and ${\widehat{\phi }}_{n}$ is the model phase. In case of a rate-only feedback loop, the (*n* + 1)-th model phase is given by

11 $$\hat{\phi }_{n + 1} = \hat{\phi }_{n} + \frac{1}{2}\left( {\dot{\phi }_{n} T + \dot{\phi }_{n + 1} T} \right),$$

where *T* is the integration time, and the dot denotes the first time derivative. With a computation delay of one update interval ${\dot{\phi }}_{n+1}$ *T* can be obtained using

12 $$\dot{\phi}_{(n + 1)} T = K_{1} \tilde{\phi }_{(n - 1)} + K_{2} \sum\limits_{(i = 1)}^{(n - 1)} {\tilde{\phi }_{i} } + K_{3} \sum\limits_{i = 1}^{n - 1} {\sum\limits_{j = 1}^{i} {\tilde{\phi }_{j} } \tilde{\phi }_{i} }$$

The coefficients ${K}_{1},{K}_{2}$, and ${K}_{3}$ characterize the tracking loop properties, such as bandwidth, dampening, etc. For the Swarm GPS receiver, the coefficients together with other parameters are given in Table 2.

**Table 2:** Third-order DPLL loop coefficients (${K}_{1},{K}_{2},{K}_{3}$) for different design bandwidths *B* based on Stephens and Thomas (1995)

${B}_{\text{DU}}$[Hz]	${K}_{1}$	${K}_{2}$	${K}_{3}$	T [s]	${\omega }_{0}$[Hz]	$a$	$b$	${B}_{\text{CU}}$[Hz]
15	0.2142	0.02208	8.655 × 10^−4^	0.01	9.5	2.43	2.25	8.5
10	0.1741	0.01313	3.585 × 10^−4^	0.01	7.1	2.60	2.45	6.5
1.00	0.1741	0.01313	3.585 × 10^−4^	0.1	0.71	2.60	2.45	0.65
0.75	0.14597	0.008619	1.8455 × 10^−4^	0.1	0.57	2.66	2.56	0.54
0.50	0.1095	0.004614	6.745 × 10^−5^	0.1	0.41	2.78	2.69	0.40
0.25	0.06253	0.001406	1.075 × 10^−5^	0.1	0.22	2.89	2.83	0.22

The values apply for rate-only numerically controlled oscillator (NCO) updates, super-critical damping, and a one-step computational delay. For comparison, continuous-update loop coefficients ${\omega }_{0}$, *a*, and *b* as defined in Ward et al. (2006) are provided for the respective update intervals *T*

### Continuous-update approximation

In the limiting case of infinitesimally small update intervals $T$, the discrete-update (DU) loop may be described by a continuous-update (CU) loop with a rational transfer function

13 $$H\left( s \right) = \frac{{{\kappa _1}{s^{ - 1}} + {\kappa _2}{s^{ - 2}} + \cdots + {\kappa _N}{s^{ - N}}}}{{1 + {\kappa _1}{s^{ - 1}} + {\kappa _2}{s^{ - 2}} + \cdots + {\kappa _N}{s^{ - N}}}}$$

of order $N=3$ in the Fourier domain, where ${\kappa }_{j}={K}_{j}/{T}^{j}$ and $s=2i\pi f$ (Stephens and Thomas 1995). This leads to the CU loop bandwidth

14 $${B_{{\text{CU}}}} = \frac{{\kappa _1^2{\kappa _2} - {\kappa _1}{\kappa _3} + \kappa _2^2}}{{4\left( {{\kappa _1}{\kappa _2} - {\kappa _3}} \right)}}$$

Following Ward et al. (2006), this may also be expressed as

15 $${B_{{\text{CU}}}} = \frac{{{\omega _0}\left( {a{b^2} + {a^2} - b} \right)}}{{4\left( {ab - 1} \right)}},$$

where $a={\kappa }_{2}/{\kappa }_{3}^{2/3}$ and $b={\kappa }_{1}/{\kappa }_{3}^{1/3}$ are bandwidth-independent filter constants that determine the damping and overshoot of the output signal in response to a step change of the input signal. The coefficient ${\omega }_{0}={\kappa }_{3}^{1/3}$ denotes the natural frequency of the filter and determines the filter bandwidth for given *a* and *b*. Typical values of these parameters are *a* = 1.1 and *b* = 2.4 (Ward et al. 2006), whereas values of 2.5–3 (Table 2) apply for the Swarm PLL in accordance with the choice of supercritical damping.

Making use of (13) and the known loop parameters, the signal $\widehat{g}\left(t\right)$ at the output of the tracking loop can be computed for a given input signal $g\left(t\right)$ by means of the Fourier-transform $F$ using the relation

16 $$\hat g\left( t \right) = {F^{ - 1}}\left( {H\left( s \right) \cdot F\left( {g\left( t \right)} \right)} \right).$$

Given $H\left(s\right)\ne 0 \forall s$, this may be inverted, such that

17 $$g\left( t \right) = {F^{ - 1}}\left( {\frac{1}{{H\left( s \right)}} \cdot F\left( {\hat g\left( t \right)} \right)} \right).$$

Use of this relation along with the Fourier-domain representation of the transfer function in (13) provides a computationally convenient and effective way of convoluting a known output signal with the inverse loop transfer function. It avoids the complexity of inverting the discrete-update transfer function in the time domain and will be used in this study for recovering the L2 tracking error based on (9).

### Empirical transfer function

**Fig. 3:**
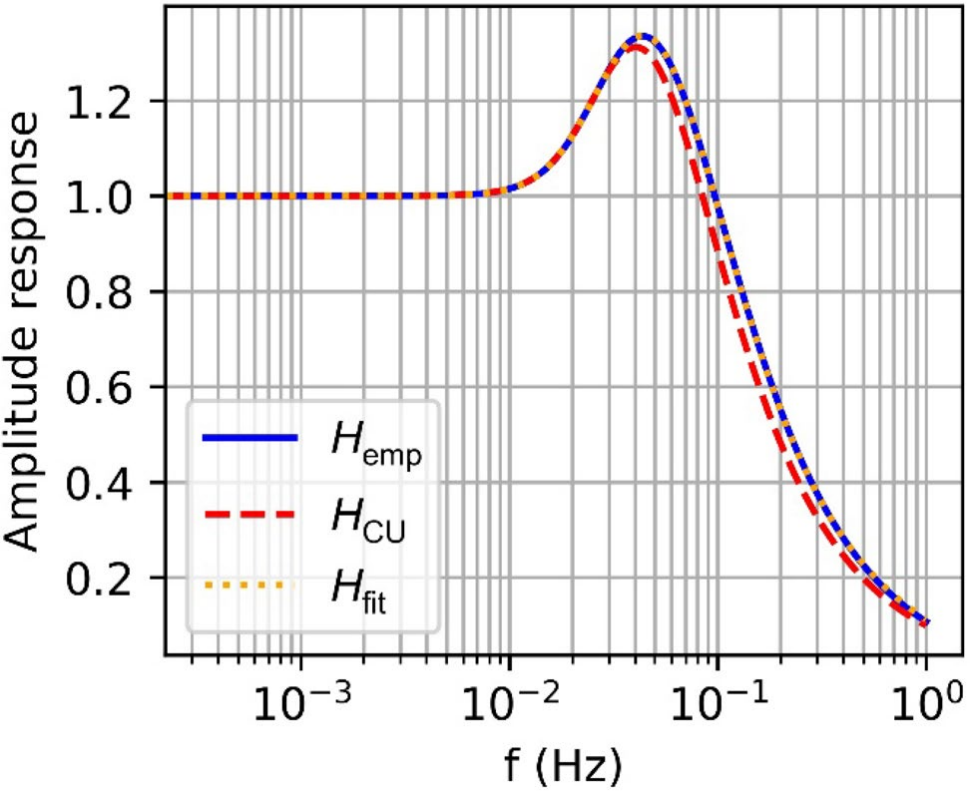
Amplitudes of the discrete-update transfer function, a 4/6-order approximation, and the continuous-update approximation for the Swarm L2 tracking loop with $B=0.25 \mathrm{Hz}$ and $T=0.1 \mathrm{s}$

**Fig. 4:**
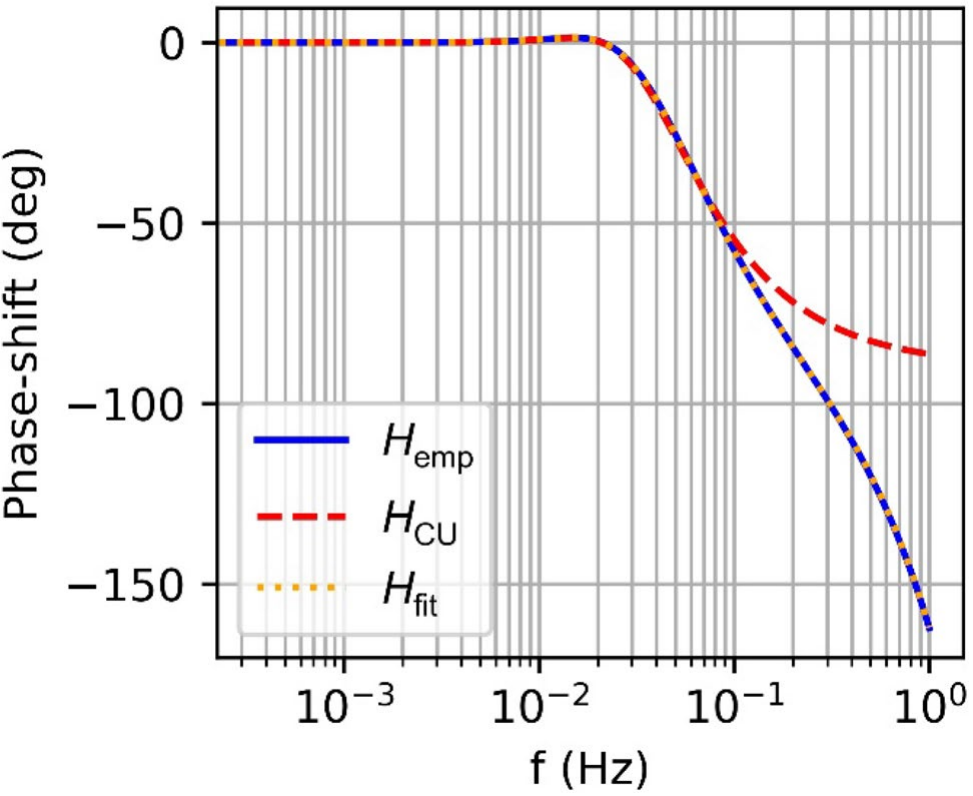
Phase shifts of the discrete-update transfer function, compared to a 4/6-order approximation, and the continuous approximation for the Swarm L2 tracking loop with $B=0.25 \mathrm{Hz}$ and $T=0.1 \mathrm{s}$

While convenient to use, the continuous-update approximation does not provide a realistic model of the actual loop behavior if the product of the bandwidth *B* and the integration time *T* violates the condition $BT \ll 1$ (in Stephens and Thomas (1995): $BT \le 0.02$) and if the loop-filter has a computation delay as it is the case for the Swarm GPS receivers. Notable deviations from the true transfer function can, in particular, be noted in the phase shift at frequencies above the characteristic frequency (see Figs. 3 and 4). Here *H*_emp_ denotes the empirical transfer function derived from an artificial signal, *H*_CU_ is the continuous update approximation and *H*_fit_ the approximated transfer function, see (18). To cope with this limitation, we evaluated the transfer function of the discrete-update loop implementation according to Fig. 2 for a signal covering the frequency range of interest. The amplitude and phase response in the frequency domain were then approximated by a rational transfer function

18 $${H_{{\text{fit}}}}\left( s \right) = \frac{{{b_2}{s^3} + {b_3}{s^2} + {b_4}s + {b_5}}}{{{s^5} + {a_1}{s^4} + {a_2}{s^3} + {a_3}{s^2} + {a_4}{s^1} + {a_5}}},$$

with an order $6$ in the denominator and order 4 in the numerator (4/6-order), that exceeds the actual loop order, and coefficients ${a}_{i}$ and ${b}_{i}$ adjusted to minimize the deviation from the actual transfer function. The specific form and order of the transfer function result from Aguirre and Hurd (1984) for a third of DPLL with computation delay. As shown in Figs. 3 and 4 for the example of the 0.25 Hz bandwidth, a good representation of the DU transfer function for frequencies of 0.001–1 Hz is obtained with a 4/6-order rational transfer function. Independent approximations of this order were determined for all relevant L2 loop settings in Table 2 and are used in the subsequent sections for correction of the corresponding phase measurements.

### Tests on synthetic data

**Fig. 5:**
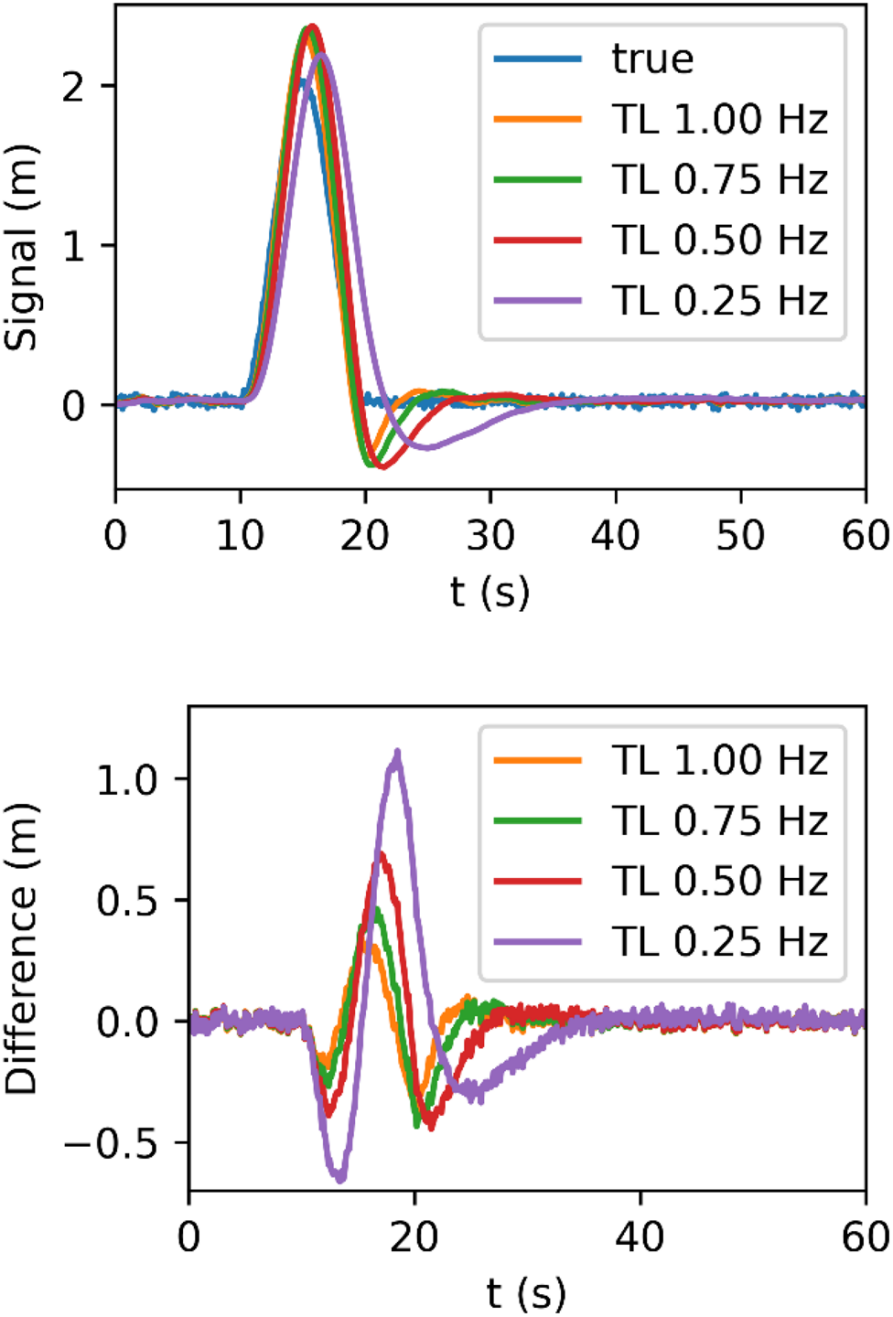
PLL response (top) and differences (bottom) for different bandwidths to a synthetic cosine-shaped pulse

To evaluate the tracking loop performance, we use a constant-zero signal with a cosine-shaped pulse of 0.1 Hz frequency and 2 m peak-to-peak amplitude starting at *T* = 10 s. In addition, white noise with a *σ* = 1 cm is added. Figure 5 shows the output of the discrete-update tracking loop at $T=0.1 {\text{s}}$ for different bandwidths as used by the Swarm receivers. As expected, Higher bandwidths result in a faster response. However, all loops overestimate the peak and need a few seconds time to follow the zero signal again. In Fig. 5 (bottom), the differences to the original signal are shown. The difference for the 0*.*25 Hz loop shows the largest amplitude, but the other bandwidths also show clear signatures. This generates systematic biases in the observations provided by the loop when encountering fast variations of the input signal.

**Fig. 6:**
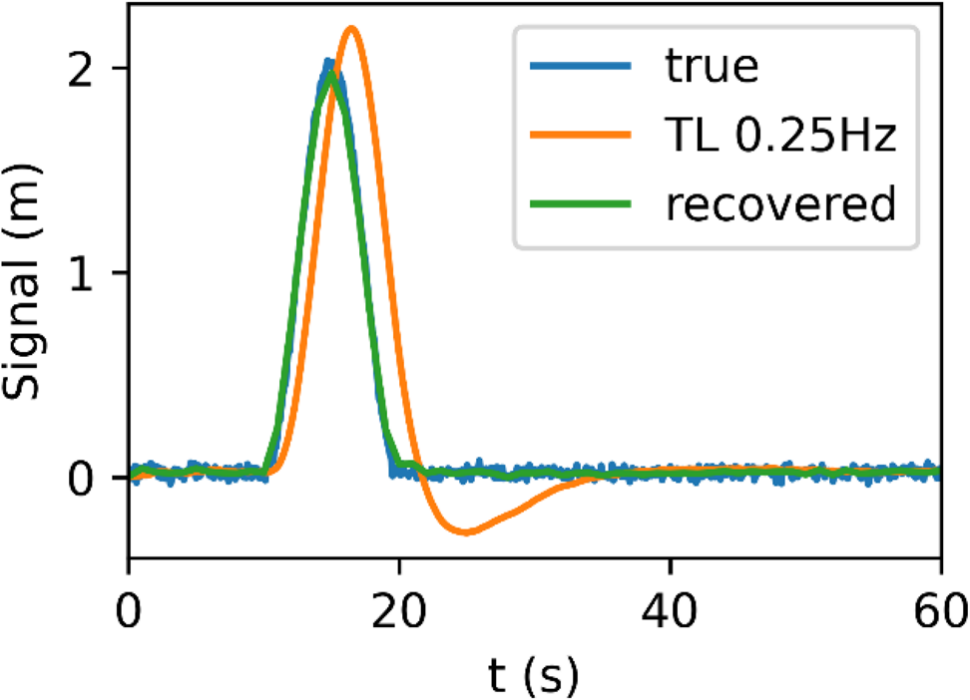
True signal, tracking loop output, and recovered values for a cosine-shaped pulse of 10 s duration and a 0.25 Hz bandwidth

When applying the inverse of the empirical transfer function derived in the previous section to the output signal with a sampling interval of 1 s, i.e., $10\bullet T$, one may recognize a close match between the original signal and the recovered signal (Fig. 6). Thus, the test demonstrates the effective use of a rational approximation of the transfer function for correcting bandwidth-related tracking errors of a discrete-update PLL, even if the measurement sampling rate is notably lower than the actual loop update rate but still comparable to the bandwidth and signal frequency.

### Application to real data

For the correction of Swarm GPS observations based on (9), we make use of the geometry-free linear combination ${\widehat{\phi }}_{\text{gf}}={\widehat{\phi }}_{2}-{\widehat{\phi }}_{1}$, where ${\widehat{\phi }}_{1}$ and ${\widehat{\phi }}_{2}$ denote the observed carrier phase range on the L1 and L2 frequencies as obtained from the RINEX observation files at a 1 s sampling. No dedicated cycle slip detection and correction is applied, since most cycle slips are already corrected in the Swarm Level 1B data (NSI 2019). However, the observation arcs were split if gaps of more than 1*.*5 s or if $|{\widehat{\phi }}_{\text{gf}}({t}_{k}) -{\widehat{\phi }}_{\text{gf}}({t}_{k+1})| /\Delta t > 1m/s$. For each of the obtained continuous phase arcs obtained this way, we will perform the inversion independently.

As discussed above, the actual discrete-update transfer function for the loop settings applicable on the day of interest was approximated by a rational function ${H}_{\text{fit}}\left(s\right)$ in the frequency domain (cf. (18)). The convolution of ${\widehat{\phi }}_{\text{gf}}$ with the inverse transfer function in (9) was then performed by multiplication of ${H}_{\text{fit}}\left(s\right)$ with the Fourier transform of ${\widehat{\phi }}_{\text{gf}}$ and back-transformation into the time domain. For application on the real phase observations, which are collected at equidistant epochs ${t}_{k}, k=0,\dots , N$, we use the Fast Fourier Transform (FFT). The phase arcs are not periodic but are assumed to be periodic by the FFT. According to Fig. 5, the main response of the loop Filter takes place within 30 s after a signal. Therefore, discontinuities at the edges need to be avoided. For that purpose, we use a polynomial fit of degree 1 to the first and last 20 s of the phase arc and extrapolate the signal 60 s at the beginning and the end. To obtain a smooth transition between the polynomial and the original signal, we blend in the first and last 10 s using a linear weighting. Eventually, we also detrend the extended signal using a linear function

19 $${\tilde \phi _{{\text{gf}}}}\left( {{t_k}} \right) = {\hat \phi _{{\text{gf}}}}\left( {{t_k}} \right) - k \cdot \left( {\frac{{{{\hat \phi }_{{\text{gf}}}}\left( {{t_{{\text{N}} + 60}}} \right) - {{\hat \phi }_{{\text{gf}}}}\left( {{t_{ - 60}}} \right)}}{{N + 120 - 1}}} \right),$$

such that no large step occurs in a periodic continuation of the signal. This transformation does not affect the loop-filter response and thus the result of (9), since a tracking loop of order three has no tracking error due to phase velocity (Ward et al. 2006). Note that the extended data vector is only used to avoid edge effects in the convolution with the inverse transfer function. Data outside the original interval are discarded after computing the L2 correction.

## Results

The precise orbit determination was performed using the development Version Bernese GNSS Software 5.3. As external products, the final GPS orbits and the high-rate 5 s GPS satellite clock corrections are used, which are provided by the Center Of Orbit Determination Europe (CODE; Dach et al. 2017). The Swarm GPS RINEX files (baseline 0401) and attitude (baseline 0401) are provided by ESA. Further inputs for the reduced dynamic orbit determination include the gravity field model EGM2008 (Pavlis et al. 2012) and the ocean tide model FES 2004 (Lyard et al. 2006). A set of constrained piecewise constant accelerations in radial, along-track, and cross-track direction is estimated at six-minute batch intervals to account for model deficiencies and non-gravitational forces. The kinematic positions are computed from undifferenced GPS carrier phase observations.

**Fig. 7:**
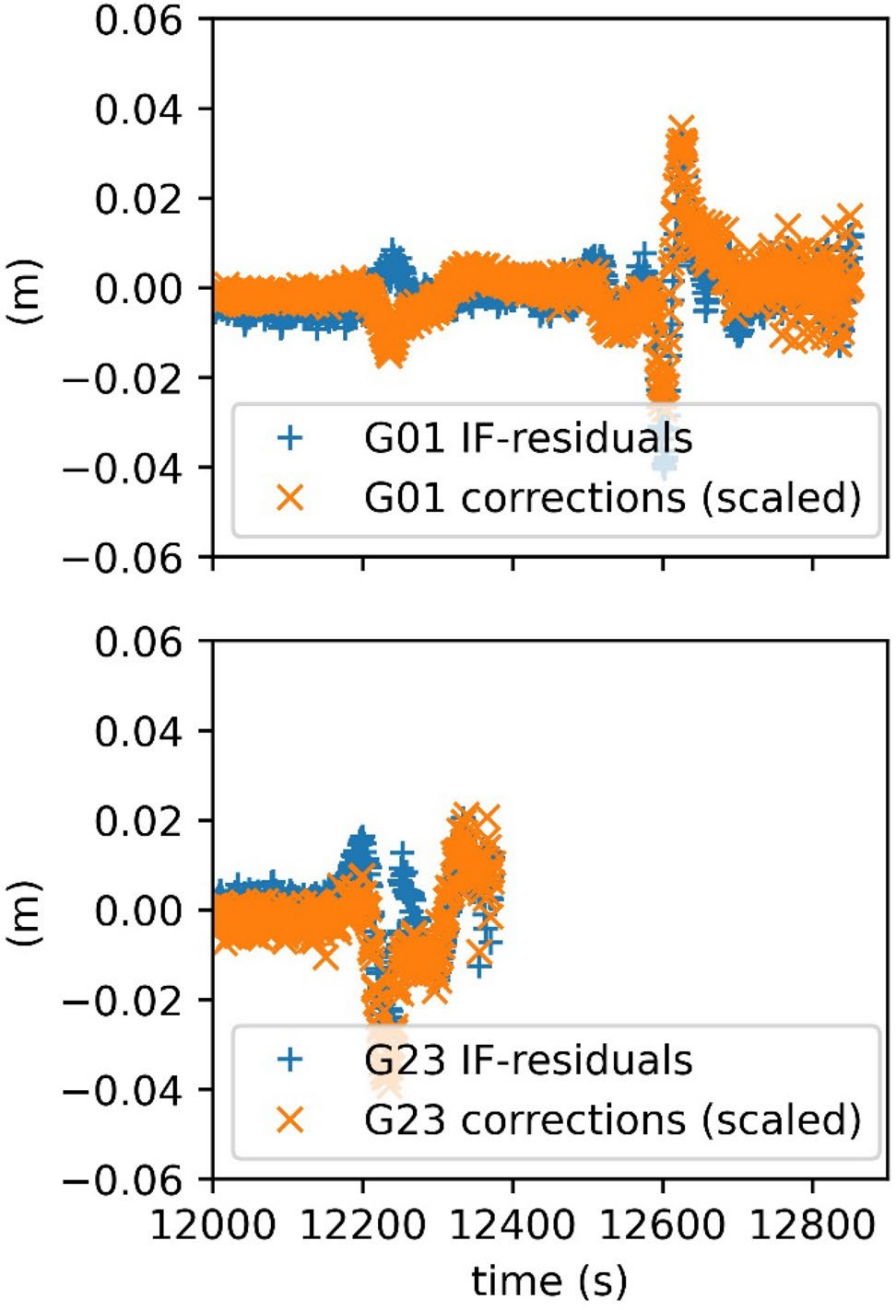
L2 corrections for Swarm A (2014/11/1) scaled to IF linear combination and IF phase residuals for GPS satellites G01 and G23 to a reduced dynamic orbit

A first estimate for the corrections needed is the ionosphere-free (IF) phase residuals to a reduced dynamic (RD) orbit and the associated receiver clock solution. Since the RD orbit shows a higher dynamical stiffness, it better represents the assumed “true” position of the satellite than the kinematic positions. At the locations where artifacts occur, the IF phase residuals may become large (Schreiter et al. 2019), but due to the estimation of epoch-wise receiver clock offsets, they have an approximately epoch-wise zero mean. Figure 7 (top) shows an example of IF phase residuals for the GPS satellite G01 and the associated measurement corrections. For comparing the corrections to the IF phase residuals, the corrections must be scaled by approx. 1.546 due to the pre-factor of ${\phi }_{2}$ in the IF linear combination. At the locations where the IF phase residuals are getting large, the corrections show very similar behavior. However, earlier (around second 12,200 s), there is an apparently opposite correction for G01, which is caused by another GPS satellite (G23). The epoch wise phase residuals are coupled by the epoch-wise receiver clock estimate. As G23 is highly affected, the receiver clock estimate is also affected, which results in a different estimated range for G01.

**Fig. 8:**
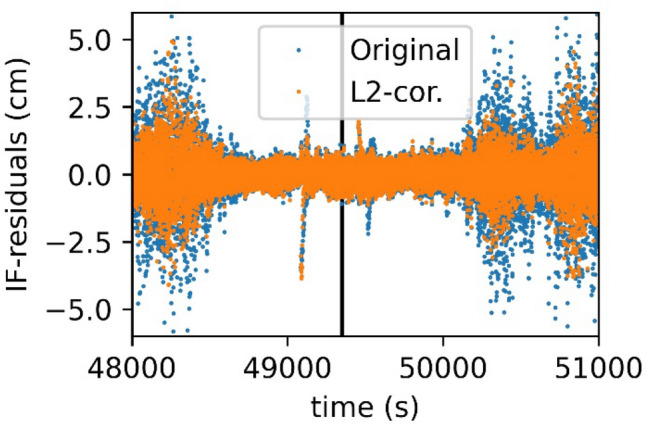
IF phase residuals before and after applying the corrections to the L2 phase for Swarm A, 2015/03/01. The black line indicates the equator crossing

After applying the corrections, the ionosphere-free phase residuals with respect to a reduced dynamic orbit are notably reduced after applying the corrections. In Fig. 8, one equator-crossing pass is displayed with the black line indicating the equatorial crossing. Around 48,000 s and 50,500 s are the polar regions, which typically show larger residuals. Near second 49,000 s and second 49,500 s larger spikes are visible. These spikes then lead to systematic differences when comparing a reduced dynamic orbit to kinematic positions. It may be recognized that after applying the corrections to the L2 phase observable, the residuals become smaller in the polar regions, but also the spikes disappear to some extent. However, the negative part of the first spike is still present. It is the beginning of a phase arc, where no reliable correction could be performed.

### Orbit quality

**Fig. 9:**
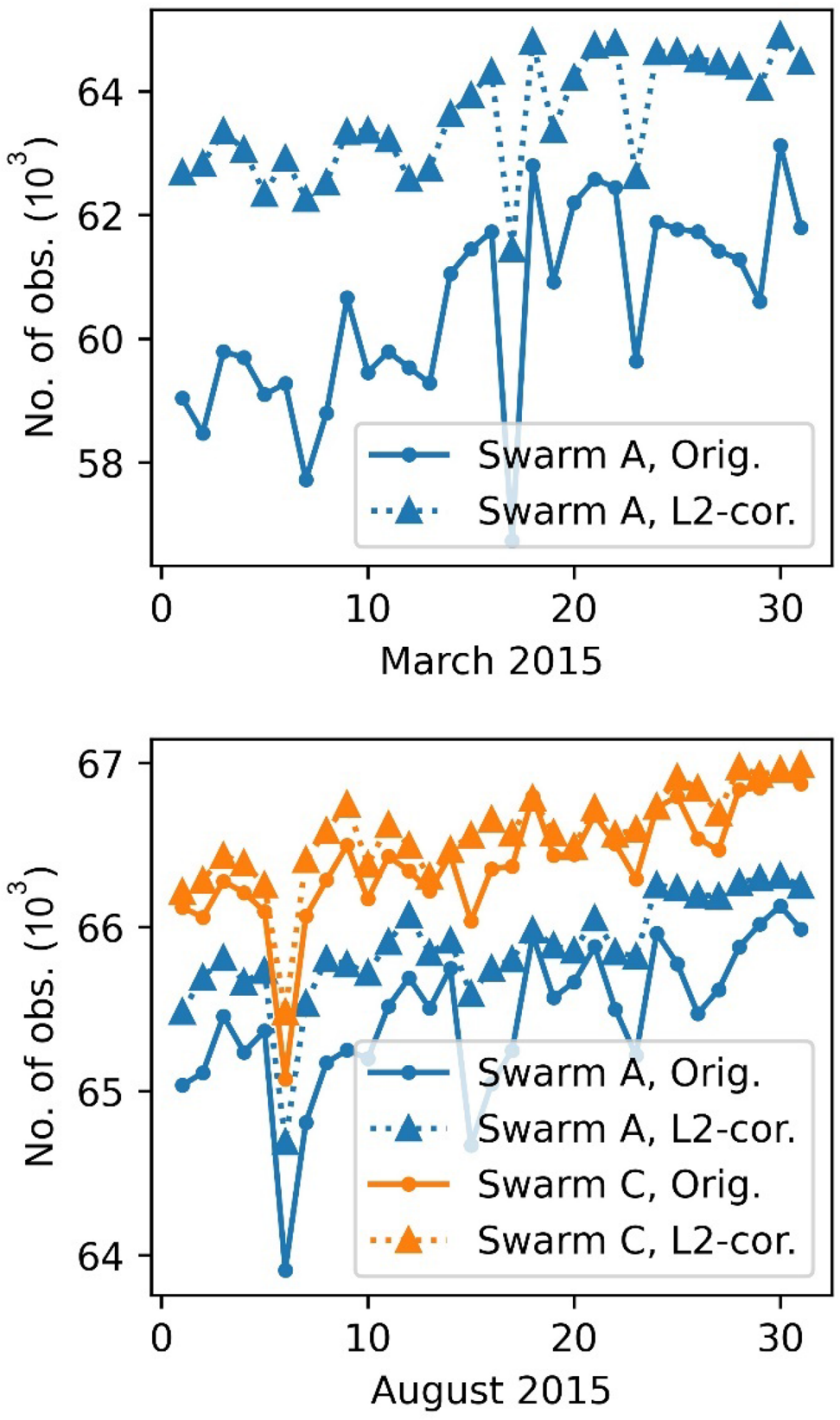
Number of observations used for reduced dynamic orbit determination after outlier screening

**Fig. 10:**
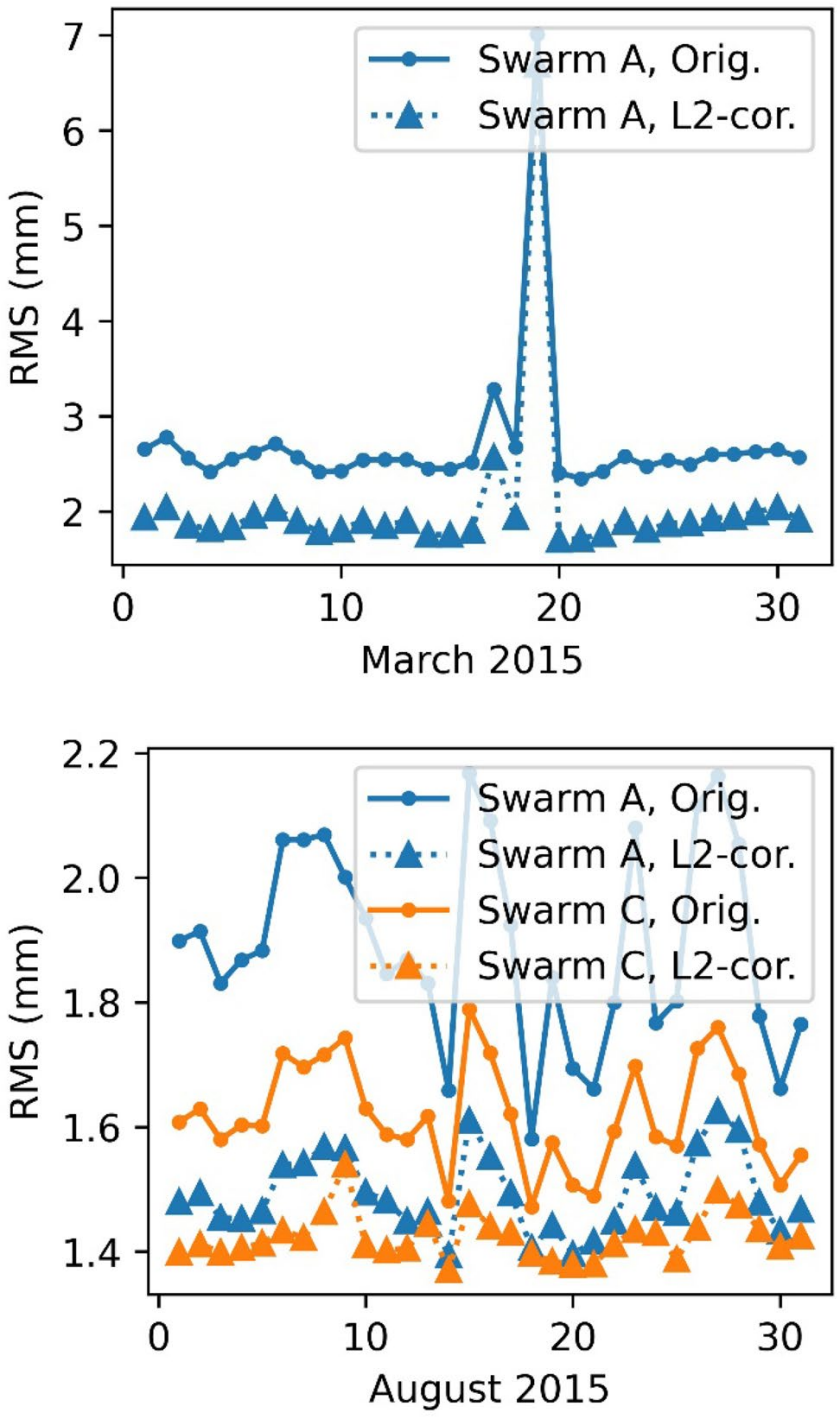
Impact of the L2 corrections on the RMS carrier phase residuals of the reduced dynamic orbit determination

**Fig. 11:**
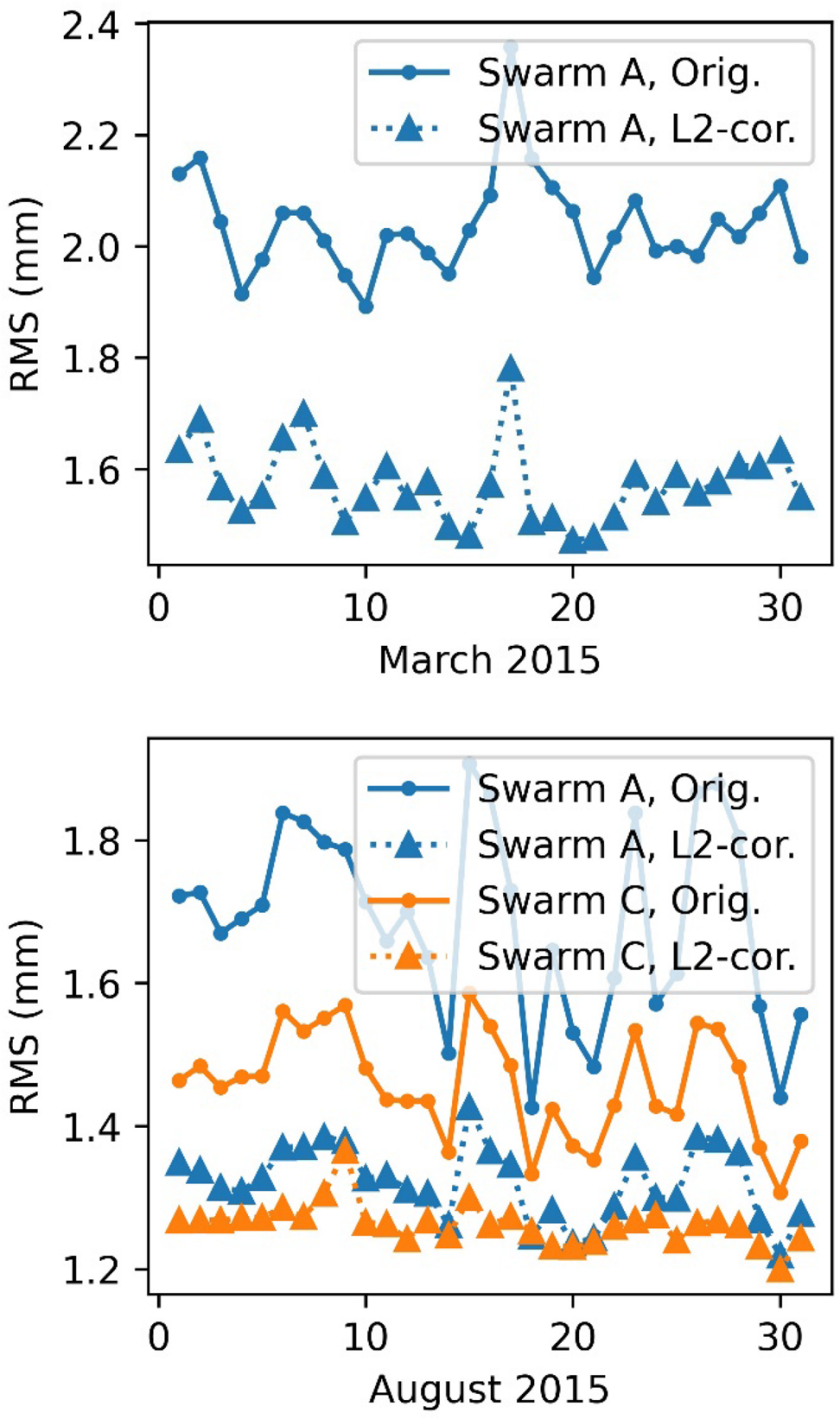
Impact of the L2 corrections on the RMS carrier phase residuals of the kinematic orbit determination

**Fig. 12:**
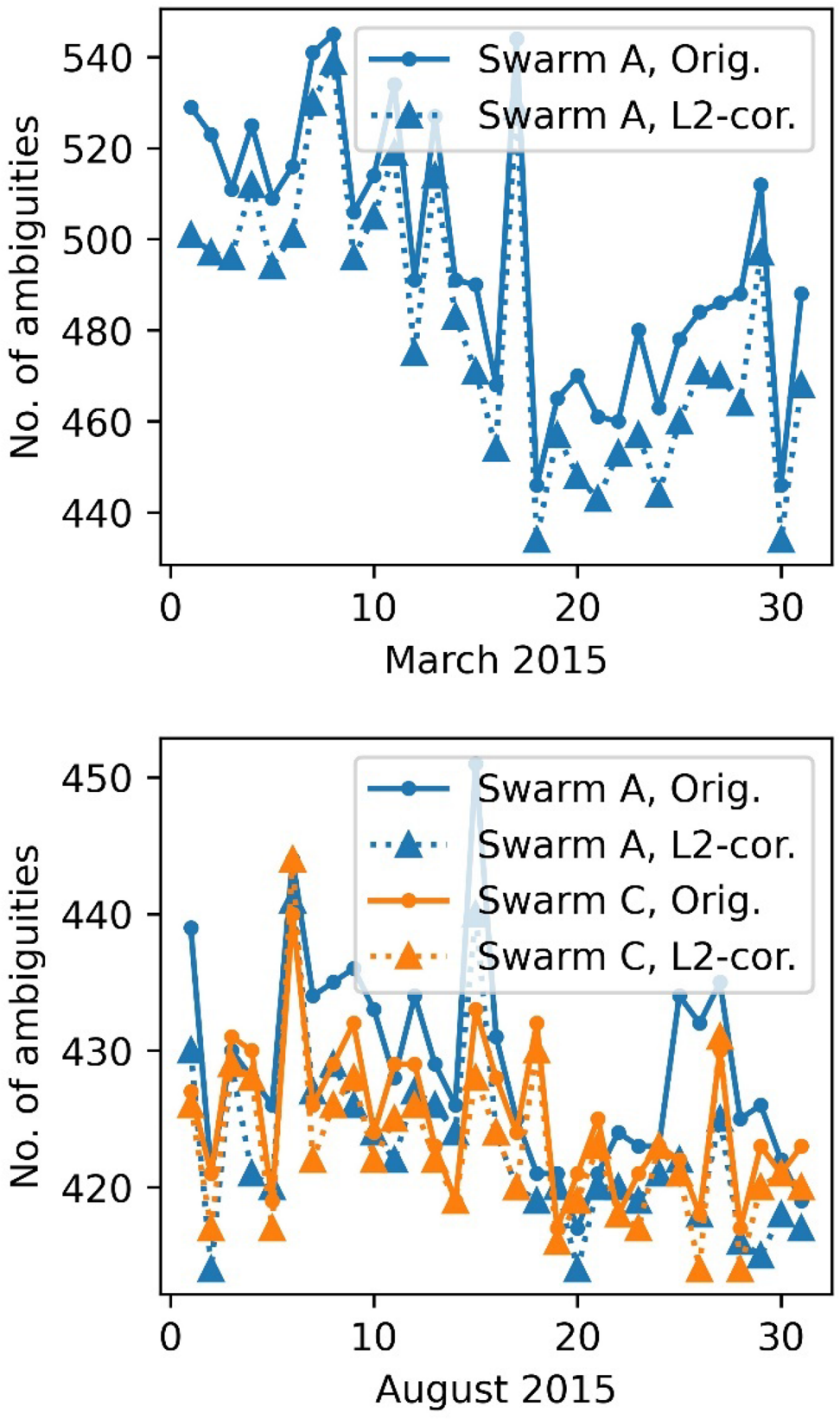
Number of ambiguities for daily arcs during precise orbit determination

To evaluate the impact on orbit fitting level, we compare the number of observations used for the final orbit determination, the post-fit root-mean-square (RMS) for both the reduced dynamic and the kinematic orbit and the number of ambiguities set for each scenario, see Figs. 9, 10, 11 and 12. It can be observed that more observations could be used for all days if the corrections to the L2 phase observable are applied. This is mostly due to smaller IF-residuals, which reduces the number of observations that are rejected in the phase screening. Also, this leads to fewer gaps and, in turn, allows for a lower number of ambiguity parameters (Fig. 12). For March 2015, up to 7% more observations could be retained. For August 2015 still up to 1% more observations are used. Figure 9 (bottom) also shows an increased number of useful observations for Swarm C that uses a two-times wider bandwidth in August 2015 than Swarm A. This increase relates to the fact that a fixed threshold is used on ionosphere-fee phase residuals in the preprocessing to screen for bad observations in all data sets. As a result of the wider bandwidth of Swarm C, systematic tracking errors and the magnitude of phase residuals are reduced compared to Swarm A. Accordingly, a larger number of observations are accepted and used for POD. The increased number of available observations due to the tracking loop updates were already observed in van den IJssel et al. (2016).

The post fit RMS could be improved from 3 mm RMS down to 2 mm RMS for the reduced dynamic orbit for March 2015. Even the very disturbed days (17.3.2015–19.3.2015) show an improvement, however, the RMS is still highly above typical levels due to a geomagnetic storm taking place on 17.3.2015 with Kp-indices up to 8− (GFZ 2019). Also, for the kinematic positioning, an improvement of the carrier phase residuals from 2.1 mm down to 1.6 mm can be observed. For August 2015 this difference is less pronounced, but still the RMS regarding the reduced dynamic orbits could be reduced from 1.9 mm to 1.5 mm for Swarm A and from 1.6 to 1.4 mm for Swarm C. For the kinematic positioning a reduction from 1.7 mm down to 1.3 mm, Swarm A, and 1.5 mm to 1.25 mm, Swarm C was achieved. Not a single day was degraded.

For an external assessment of the orbit quality, we make use of Satellite Laser Ranging (SLR) validation Arnold et al. (2018). Even though the orbits were processed using the ITRF2008-compatible reference frame realization IGb08, we use the SRLF2014 instead of the SLRF2008. This approach was proven to be beneficial in Arnold et al. (2018) because of information for post seismic deformation and improved station coordinates. We use a subset of 12 of the available SLR stations: 7090, 7105, 7119, 7501, 7810, 7825, 7827, 7839, 7840, 7841, 7941, and 8834. Among these stations are Herstmonceux, Graz, Greenbelt, Mount Stromlo, Yarragadee, and Zimmerwald, which are known for particularly high quality and amount of SLR observations. An outlier threshold of 200 mm is used. A selection of stations near the geomagnetic equator could not be used due to the very limited number of active SLR stations in that region. For the reduced dynamic orbits in March 2015, the results are given in Tables 3 and 4. Here, both the mean value and standard deviation are reduced by 0.5–1.0 mm, and the RMS SLR residuals decrease by about 1.2 mm. For the kinematic orbit standard deviation and RMS also improves in the corrected scenario, the offset drops by 0.9 mm. The standard deviation and the RMS are reduced by approximately 3.6 mm. For August 2015, in Tables 5 and 6, no large differences can be observed in the reduced dynamic scenario. However, for the kinematic orbits, the mean offset is reduced by 0.2 mm to 0.3 mm. Only minor improvements at the sub-millimeter scale can be observed in RMS and standard deviation.

**Table 3:** SLR residuals for reduced dynamic orbit for March 2015

Scenario	Sat	Number of observations	Mean (mm)	std (mm)	RMS (mm)
Original	A	1433	4.93	26.09	26.54
L2-correction	A	1433	4.34	25.05	25.32

**Table 4:** SLR residuals of kinematic orbits for March 2015

Scenario	Sat	Number of observations	Mean (mm)	std (mm)	RMS (mm)
Original	A	1408	2.47	30.02	30.12
L2-correction	A	1408	1.29	26.46	26.48

**Table 5:** SLR residuals of reduced dynamic orbits for August 2015

Scenario	Sat	Number of observations	Mean (mm)	std (mm)	RMS (mm)
Original	A	1775	7.01	14.23	15.86
L2-correction	A	1775	6.78	14.33	15.85
Original	C	2047	5.00	14.67	15.49
L2-correction	C	2047	5.00	14.75	15.57

**Table 6:** SLR residuals of kinematic orbits for August 2015

Scenario	sat	Number of observations	Mean (mm)	std (mm)	RMS (mm)
Original	A	1775	4.95	18.75	19.39
L2-correction	A	1775	4.63	18.48	19.04
Original	C	2047	4.39	16.54	17.11
L2-correction	C	2047	4.21	16.37	16.90

For August 2015, the ionospheric activity was much lower, see Fig. 1, and the updates of the L2 PLL were already performed (Table 1). Also, the lack of SLR stations near the geomagnetic equator weakens the capability to validate possible orbit improvements in that region.

### Gravity field solutions

**Fig. 13:**
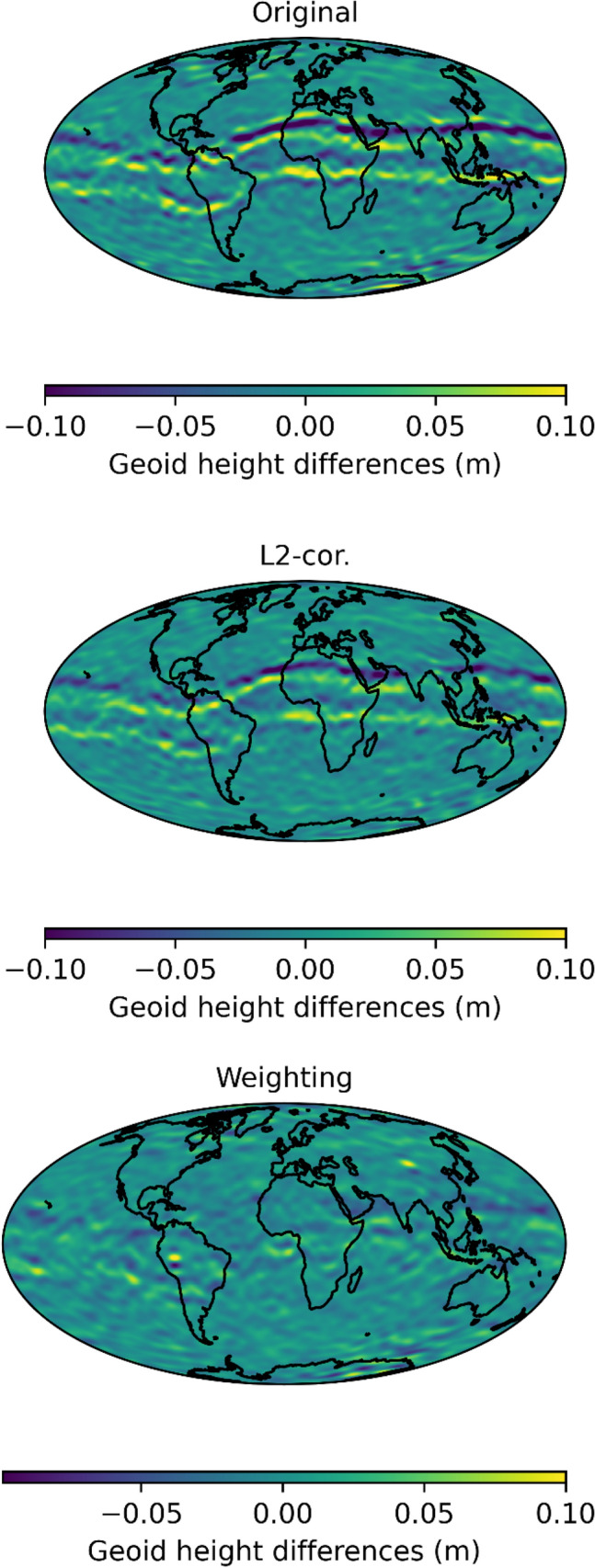
Geoid height differences compared to the monthly JPL-RL06 GRACE gravity field solution for March 2015

**Fig. 14:**
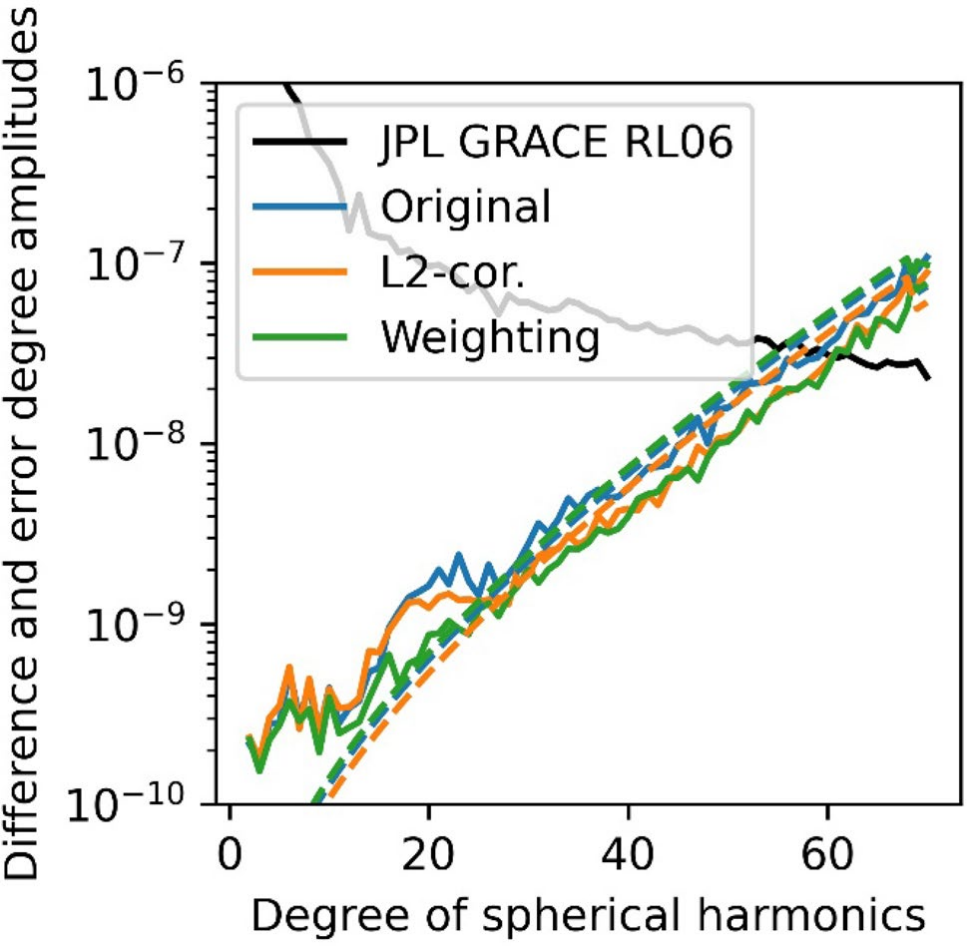
Difference (solid) and error (dashed) degree amplitude compared to the monthly JPL-RL06 GRACE gravity field solution

The gravity field solutions were computed using the celestial mechanics approach (Beutler et al. 2010). Further, we follow the procedure outlined in Jäggi et al. (2016). In the celestial mechanics approach, we first fit an a priori orbit to the kinematic positions considering the EGM2008 gravity field model and the ocean tide model FES 2004. Daily arcs are computed with 15 min. empirical piecewise constant accelerations to compensate for deficiencies in the orbit model. Normal equations for the orbit and gravity field parameters are set up on a daily basis, using again the kinematic positions as pseudo observations. We set up the normal equations for each day and pre-eliminated the orbital parameters and empirical accelerations. Then, we stack the normal equations of one month and solve for the gravity field coefficients. To evaluate the gravity field solutions, we compare to the monthly JPL-RL06 GRACE gravity field solution (Bettadpur 2018; GRACE 2018). First, we perform a visual inspection of the resulting geoid height differences, smoothed using a Gaussian filter with a radius of 400 km, checking if the equatorial artifact is mitigated and if other artifacts occur (see Fig. 13). The correction scenario is capable of reducing the equatorial artifact to a limited extent. We also compare to a solution obtained in a previous study using weighting of observations (Schreiter et al. 2019). Still, the equatorial artifact is least visible in the weighting solution. However, the noise patterns in the polar regions are less pronounced when correcting L2 measurements based on the inverse loop transfer function. In the weighting approach, observations with loop-related tracking errors are notably down-weighted, which leads to large co-variances in the kinematic positions and weakens their impact on the gravity field solution. Using the kinematic positions based on the corrected observations, these positions still have a similar weight in the gravity field solution as in the original one, since the co-variance given for the positions mostly represents the geometry (Jäggi et al. 2011). In the difference and error degree amplitudes, Fig. 14, the corrected observations lead to a reduction of the difference w.r.t. the reference gravity fields and show the smallest error degree variances (dashed line). However, still, the difference amplitudes are significantly smaller using the weighting strategy. Due to insignificant differences, this plot is not displayed for August 2015.

The solution based on L2 phase corrections and the solution based on data weighting show an improvement compared to the original solution (Fig. 14). Formal errors are smaller at all degrees when using the corrected L2 data as compared to the original data due to a larger number of accepted observations. For the actual errors, the benefit of the L2 correction can primarily be seen in higher degrees (> 10). However, this may be expected, as the correction mostly affects frequencies near 1/30 Hz, which corresponds to a few hundred kilometers in spatial resolution and is therefore invisible in the low degrees, which cover larger scales. In the very low degrees, the weighting scenario is slightly better, whereas in the higher degrees, the L2 correction scenario and the weighting solution become comparable. Comparing the RMS of geoid height differences weighted with the cosine of the latitude (wRMS) for March 2015, see Table 7, again the weighting solution shows the lowest value with 12.13 mm compared to the monthly GRACE JPL-RL06 gravity field solution. Still, the best fit with respect to the kinematic positions and the maximum number of kinematic positions used are obtained in the correction scenario. When comparing the results for August 2015, see Table 8, one may see that the wRMS of 8.8 mm, almost matches the value from the weighted solution, 8.5 mm, for Swarm A, but for Swarm C the weighted RMS is 8.8 mm for the weighted solution and 8.6 mm for the unmasked correction. In the wSTD over the oceans, the corrected scenario shows the smallest value. The number of positions used is similar in all cases, but the best fit with respect to the kinematic positions could be obtained using the correction.

**Table 7:** Gravity field comparison for March 2015. The monthly GRACE JPL-RL06 gravity field is used as reference

Scenario	wRMS (monthly) mm	wSTD (monthly) mm	No. kin pos	RMS kin. pos mm
A Original	21.42	28.25	695,673	2.61
A L2-correction	17.17	26.85	761,586	2.27
A Weighting	11.67	23.06	706,698	2.58

For the wSTD over the oceans the resolution is limited to degree and order 20. Weighting was performed using the second derivative combined with the Rate Of TEC Index (ROTI; Schreiter et al. 2019)

**Table 8:** Gravity field comparison for August 2015. The monthly GRACE JPL-RL06 gravity field is used as reference

Scenario	wRMS (monthly) mm	wSTD (monthly) mm	No. kin. pos	RMS kin. pos mm
A Original	9.56	20.65	796,761	1.97
A L2-correction	8.79	20.54	801,294	1.59
A Weighting	8.51	20.98	799,398	2.05
C Original	8.40	20.27	801,576	1.70
C L2-correction	8.71	20.11	802,515	1.52
C Weighting	8.76	20.52	802,695	1.78

## Summary and Conclusions

The limited bandwidth for L2 carrier phase tracking in the Swarm GPS receiver is responsible for increased carrier phase errors occurring near the geomagnetic equator and related artifacts in kinematic position solutions and gravity field models. Whenever the tracked GPS signals are subject to rapidly changing ionospheric path delays, the loop filter introduces systematic biases in the measured L2 phase, whereas the L1 phase is essentially unaffected due to much higher bandwidth. Based on the L2 PLL design knowledge, the loop response can be modeled, and the L2 tracking error can be recovered through convolution of the measured L1–L2 phase difference with the inverse loop transfer function. In this way, corrected L2 observations can be obtained, which are, ideally, free of systematic errors. In practice, the application of the concept suffers from various limitations. First of all, 10 Hz carrier phase observations would be required for the rigorous inversion of a discrete loop with 100 ms update rate rather than the 1 Hz measurements made available in the Swarm mission. Secondly, the finite length of continuous tracking arcs results in edge effects, which limit the quality of the derived phase corrections near the start and end of the track. Despite these limitations, a partial reconstruction of the true L2 phase is possible and helps to improve the overall data quality.

Considering periods of high ionospheric activity and narrow loop bandwidth, it is shown that a reduction of carrier phase residuals from 3 mm RMS to 2 mm RMS can be achieved in the reduced dynamic orbit of the Swarm satellites using the proposed correction. Likewise, the artifacts in the gravity field solution around the geomagnetic equator are mitigated, and the quality of the gravity field in the polar regions can be improved. Compared to the down-weighting of erroneous observations used in earlier studies of Swarm gravity field determination, the present correction of loop-related carrier phase errors still shows a larger amplitude of gravity field errors near the equator. However, instead of rejecting or down-weighting observations, the observations can, at least partly, be reconstructed, and a larger number of observations is thus retained. If desired, a combination of a priori loop error corrections with a suitably tuned weighting scheme may be desired for optimum gravity field recovery in future analyses.

Since the systematic errors in the L2 phase observable also affect derived products, for example TEC, a correction should also be considered for other purposes than precise orbit determination. Since the equatorial artifact in GPS-only gravity fields is also known from the GOCE mission, it would be beneficial to know the TL settings for other LEO satellites to investigate if there are also significant artifacts in GPS-only orbits and, if possible, to correct them.

## Data Availability

Swarm related data products are provided by ESA (http://swarm-diss.eo.esa.int). Auxiliary products used for precise orbit determination are provided by the IGS via anonymous ftp (http://ftp.aiub.unibe.ch/). The GRACE gravity field solutions are provided via ICGEM (http://icgem.gfz-potsdam.de/home). For precise orbit determination and gravity determination the development version 5.3 of the Bernese GNSS software was used, which is not publicly available.
